# GSA STUDENT CHAPTERS: NEW OPPORTUNITIES FOR US AND INTERNATIONAL HIGHER EDUCATION INSTITUTIONS

**DOI:** 10.1093/geroni/igad104.0602

**Published:** 2023-12-21

**Authors:** Lauren Bouchard, Marilyn Gugliucci, Terri Harvath

## Abstract

The Gerontological Society of America (GSA) Board of Directors approved the formation of a committee to explore the creation of GSA Student Chapters in their December 2020 board meeting. After 13 meetings, a committee wrote a GSA Student Chapter guide outlining policies and procedures for implementation and operation. Next, a GSA Student Chapter pilot project was implemented during the 2022-23 academic year at nine higher education institutions (HEIs) -- eight in the US and one in Portugal. This session will provide the background of (1) how the GSA Student Chapter project began including its design and implementation process, (2) how a pilot GSA Chapter maneuvered bringing together their Sigma Phi Omega Chapter, DreamCatchers, and GSA Student Chapter under a “Gerontology Club” umbrella, (3) how another chapter developed the chapter across multiple campuses, (4) the implementation of a GSA Student Chapter that is housed in a medical school, (5) how a GSA Student Chapter formed in a non-U.S. country, (6) how to connect a student chapter in the U.S. with a chapter that is in another country. A discussant will conclude this session, and there will be time for a question and answer session.

